# Cross-sectional and longitudinal associations between energy intake and BMI z-score in European children

**DOI:** 10.1186/s12966-016-0344-3

**Published:** 2016-02-16

**Authors:** Antje Hebestreit, Gianvincenzo Barba, Stefaan De Henauw, Gabriele Eiben, Charalampos Hadjigeorgiou, Éva Kovács, Vittorio Krogh, Luis A. Moreno, Valeria Pala, Toomas Veidebaum, Maike Wolters, Claudia Börnhorst, on behalf of the IDEFICS Consortium

**Affiliations:** Leibniz-Institute for Prevention Research and Epidemiology – BIPS GmbH, Achterstr. 30, D-28359 Bremen, Germany; Institute of Food Sciences, National Research Council, Avellino, Italy; Department of Public Health, Ghent University, Ghent, Belgium; Department of Public Health and Community Medicine, University of Gothenburg, Gothenburg, Sweden; Research and Education Institute of Child Health, Strovolos, Cyprus; Department of Pediatrics, University of Pécs, Pécs, Hungary and Institute for Medical Information Processing, Biometrics and Epidemiology and German Centre for Vertigo and Balance Disorders, Ludwig Maximilian University, Munich, Germany; Department of Preventive and Predictive Medicine, Fondazione IRCCS Istituto Nazionale dei Tumori, Milan, Italy; GENUD (Growth, Exercise, Nutrition and Development) Research Group, Faculty of Health Sciences, University of Zaragoza, Zaragoza, Spain; Department of Chronic Diseases, National Institute for Health Development, Tallinn, Estonia

**Keywords:** Residual energy intake, Europe, Cohort, BMI z-score, Children

## Abstract

**Background:**

Evidence for the effect of dietary energy on BMI z-scores in young children is limited. We aim to investigate cross-sectional and longitudinal effects of daily energy intake (EI) on BMI z-scores of European boys and girls considering growth-related height dependencies of EI using residual EI.

**Methods:**

To investigate cross-sectional and longitudinal effects of daily energy intake (EI) on BMI z-scores of European boys and girls considering growth-related height dependencies of EI using residual EI.

**Methods:**

Subjects were children aged 2- < 10 y old (N = 2753, 48.2 % girls) participating in the IDEFICS (Identification and prevention of Dietary- and lifestyle-induced health EFfects In Children and infantS) baseline and follow-up examination. Usual EI (kcal/day) was calculated based on the National Cancer Institute-method excluding subjects with implausible reported EI. Effect of age, height and sex-adjusted residuals of EI on BMI z-score was investigated stratified by baseline age –group (2- < 4 y, 4- < 6 y, 6- < 8 y and 8- < 10 y) cross-sectionally using linear regression models adjusted for relevant confounders (crude model: age, sex, country; fully adjusted model: plus parental ISCED level, parental BMI, screen time; subgroup analysis: plus objectively measured physical activity). Longitudinal associations were estimated between changes in (Δ) residual EI per year and ΔBMI z-score per year with adjustments analogously to the cross-sectional models but with additional adjustment for residual EI at baseline.

**Results:**

Cross-sectionally, positive associations were observed between residual EI and BMI z-score for the full study sample, for boys and in older (≥6 years) but not in younger children in the crude and fully adjusted model. Longitudinally, small positive associations were observed between Δresidual EI per y on ΔBMI z-score per y for the full study sample and in 4- < 6 y olds in the crude and fully adjusted model.

**Conclusion:**

In conclusion, EI above the average intakes for a certain sex, age and height are weakly associated with BMI z-scores in European children. Residual EI may be considered as a useful exposure measure in children as it accounts for growth-related changes in usual EI during childhood.

**Electronic supplementary material:**

The online version of this article (doi:10.1186/s12966-016-0344-3) contains supplementary material, which is available to authorized users.

## Background

The worldwide prevalence of overweight and obesity in pre-school children and school-aged children has become a major public health concern [1]. Recently the IDEFICS (Identification and Prevention of Dietary- and Lifestyle-induced Health Effects in Children and Infants) study reported an obesity prevalence of 7 % and an overweight prevalence of 13 % among European children under 10 y of age [2]. Childhood overweight is a likely consequence of the modern environment and lifestyle, characterized by increasing sedentary activities and less physical activity (PA) as well as higher energy intakes (EI) [3, 4]. Cross-sectional studies in children and adolescents found associations between EI and Body Mass Index (BMI) z-score [5, 6].

Data on longitudinal associations between energy intake and weight development in pre-school children and school children are scarce and inconsistent. Total EI (kcal) was found to be associated with weight gain in 3 to 5 y old U.S. children [7], whereas EI was unrelated to BMI in two other U.S. studies among 8 to 14 y olds [8] and ~ 11 y old adolescents [9]. Associations between EI and weight change were observed in Italian children aged 8 y at baseline and 14 y at follow-up, but associations disappeared after adjusting for parental obesity [10]. In a Danish study, EI was related to weight gain in 8 - 10 y old overweight boys, but not in girls [11]. In adolescents EI was found to be influenced by PA, whereas PA was associated with lower body fat mass/BMI. This indicates that energy expenditure through PA counteracts greater fat mass/BMI while EI is high [8, 12]. Studying associations between reported energy intake and health outcomes is challenging, especially in children. Measurement errors resulting e.g. portion size estimation, erroneous food composition tables, incomplete recalls, misreporting or daily variations in intake pose a great problem to nutritional epidemiologists [13]. In children, dietary assessment is even more complicated as data typically rely on proxy-reporters. In addition, EI in childhood and youth is influenced by energy demands and varies over time depending on growth [14] such that absolute EI values cannot be directly compared between children of different ages.

In the light of the above and knowing that dietary habits may track from childhood into adulthood [15, 16] it is important to understand to which extent early EI is associated with changes in BMI among pre-school children. The present study aims to investigate cross-sectional and longitudinal associations between usual daily EI and (changes in) BMI z-scores of European boys and girls, accounting for growth-related changes in usual EI during childhood and trying to address the abovementioned methodological challenges.

## Methods

Data for the present cross-sectional and longitudinal study were obtained from the IDEFICS (Identification and prevention of Dietary- and lifestyle-induced health EFfects In Children and infantS) Study baseline examination (at time T0) in 2007/2008, and from the follow-up survey (T1) in 2009/2010.

### Study participants

The IDEFICS study was designed as a prospective cohort study. Children from Sweden, Germany, Hungary, Italy, Cyprus, Spain, Belgium, Estonia aged 2 to <10 y who attended selected pre-schools or kindergartens and primary schools (grades 1 and 2) were eligible for participation [17]. The children were approached via schools and kindergartens to facilitate equal enrollment of all social groups. In addition to the signed informed consent given by parents, each child was asked to give verbal assent immediately before examination. Each participating center obtained ethical approval from the local responsible authorities in accordance with the ethical standards laid down in the 1964 Declaration of Helsinki and its later amendments. All 16,228 children in the defined age categories who fulfilled the inclusion criteria at baseline were invited for follow-up examination.

### Questionnaires and anthropometric measurements

Questionnaires were developed in English, translated into local languages, and then back-translated to check for translation errors. Parents completed a questionnaire to assess - among others – parental BMI, behavioral factors and their determinants, socio-demographic characteristics and information on children’s total daily screen time (time of computer and/or TV use). For the present investigation, the highest educational level of the parents according to the International Standard Classification of Education (ISCED) [18] was used as a proxy indicator for socio-economic status (SES) of the family.

The field methods comprised anthropometric measurements of standing height (cm) using a Seca 225 stadiometer (Seca GmbH & KG, Birmingham, UK) in accordance with international standards for anthropometric assessment and weight (kg) [19]. Body weight was assessed in fasting children using a prototype of the TANITA BC 420 SMA digital scale (TANITA Europe GmbH, Sindelfingen, Germany) specifically adapted for children’s feet. All measurements were performed in light clothing (e.g. underwear). All anthropometric measurements followed detailed standard operation procedures [20].

### BMI z-scores

BMI was calculated by dividing body weight in kilograms by squared body height in meters. BMI was transformed to an age- and sex-specific z–score according to Cole et al. [21]. Weight groups (thin/normal and overweight/ obese) were categorized using age- and sex-specific cut-off values based on the extended IOTF criteria [22]. The annual change in BMI z-score (ΔBMI z-score) was calculated as BMI z-score at follow-up minus BMI z-score at baseline divided by the time span between baseline and follow-up:

$$ \Delta BMI\ \mathrm{z}\hbox{-} \mathrm{score}\kern0.5em =\kern0.5em \left(\mathrm{B}\mathrm{M}\mathrm{I}\ \mathrm{z}\hbox{-} \mathrm{score}\ \mathrm{T}1\kern0.5em \hbox{--} \kern0.5em \mathrm{B}\mathrm{M}\mathrm{I}\ \mathrm{z}\hbox{-} \mathrm{score}\ \mathrm{T}0\right)\kern0.5em /\kern0.5em \left(\mathrm{age}\ \mathrm{T}1\kern0.5em \hbox{--} \kern0.5em \mathrm{age}\ \mathrm{T}0\right). $$

### Assessment of physical activity

Free-living PA was objectively assessed using Actigraph uniaxial accelerometers (either ActiTrainer or GT1M; Actigraph, LLC, Pensacola, FL, USA), containing both identical sensor units. The monitor was set to record PA in a 15-second sampling interval (“epoch”). Accelerometer measurements were included from children who wore the accelerometer for at least three days, with at least 6 hours per day, including one weekend day. Accelerometers were mounted on the right hip of each child secured by an elastic belt. The duration of moderate-to-vigorous physical activity (MVPA) was determined according to the cut-offs of Evenson [23] as described for the IDEFICS cohort in Konstabel et al. [24].

### Dietary information

Dietary intake of the previous 24 hours was assessed using the computer-assisted 24-hour dietary recalls (24HDR), called ‘Self-Administered Children and Infant Nutrition Assessment’ (SACINA) [25]. The software was based on the YANA-C (‘Young adolescents’ nutrition assessment on computer’) system [26] developed within the HELENA Study (http://www.helenastudy.com). Proxy respondents were asked to recall the children’s diet and enter type and amount of all drinks and foods consumed during the previous day, starting with the first intake after waking up in the morning. Proxies were requested to complete repeated 24HDR, including two working days and one weekend day. Standardized photographs were used to assist accurate estimation of portion size and proxies could ask for assistance of the survey dietician when completing the 24HDR. During the recall, impossible and implausible values (e.g. energy, amount (portion size), quantity (numbers of portions), missing meals) were checked for single food items and for single recalls and were re-confirmed with the participant directly. Further, the system checked for frequent and easily forgettable foods and food combinations. Meals, drinks and snacks consumed in school or kindergarten the day prior to the 24HDR were recorded using a standardized observer sheet, completed by trained personnel.

Country-specific food composition tables (FCT) were used to match simple foods or European homogeneous multi-ingredient food items [25]. All nutrients and energy values were expressed per 100 g edible portion according to McCance and Widdowson [27]. The metabolizable energy values of foods and beverages were given in kilocalories (kcal).

The validity of proxy-reported energy intakes from the 24HDR was investigated based on comparison with total energy expenditure measured using the doubly labelled water (DLW) technique. The instrument was found to be valid to assess EI on group level rather than on individual level [28].

### Dietary data analysis

Missing quantities or implausible values (above median + 2.5 standard deviation for single food items) that could not be corrected were imputed by country, food group and age-specific median intakes (0.01 % of the entries). Incomplete 24HDR and those with four or more imputed values were excluded from the analysis. The amount of all consumed foods and beverages was recorded in grams (g).

Age and sex-specific Goldberg cut-offs adapted for use in children were applied to classify subjects with under reported, plausible reported and over reported EI as described earlier [13]. We excluded 1221 subjects classified as under-reporters (656 at T0 and 565 at T1) and 462 over-reporters (261 at T0 and 201 at T1) from the analysis.

After exclusion of misreports, individual daily energy intakes (EI) were estimated based on the National Cancer Institute Method [29] accounting for the daily variation in diet, and adjusting for weekend days/weekdays, interview sequence, age and sex. To correct for the daily variation of EI, all repeated 24HDR that were available for T0 and T1 examinations in a subgroup of children were used. Based on all IDEFICS children providing at least one plausible 24HDR, 2068 children at T0 and 903 children at T1, provided more than one 24HDR (up to 6).

### Covariate information

Age, height, sex, country of residence and - according to previous literature - total screen time per week and physical activity [10, 30, 31], parental ISCED level [32], and parental BMI [33] and MVPA were considered being potential confounders in the present study. Total television (TV) and computer use time will hereafter be termed *screen time*. Where baseline covariate information was missing, but follow-up information available, the baseline value was imputed by the follow-up value so as not to further reduce the study sample.

### Inclusion criteria for study sample

**Fig. 1:**
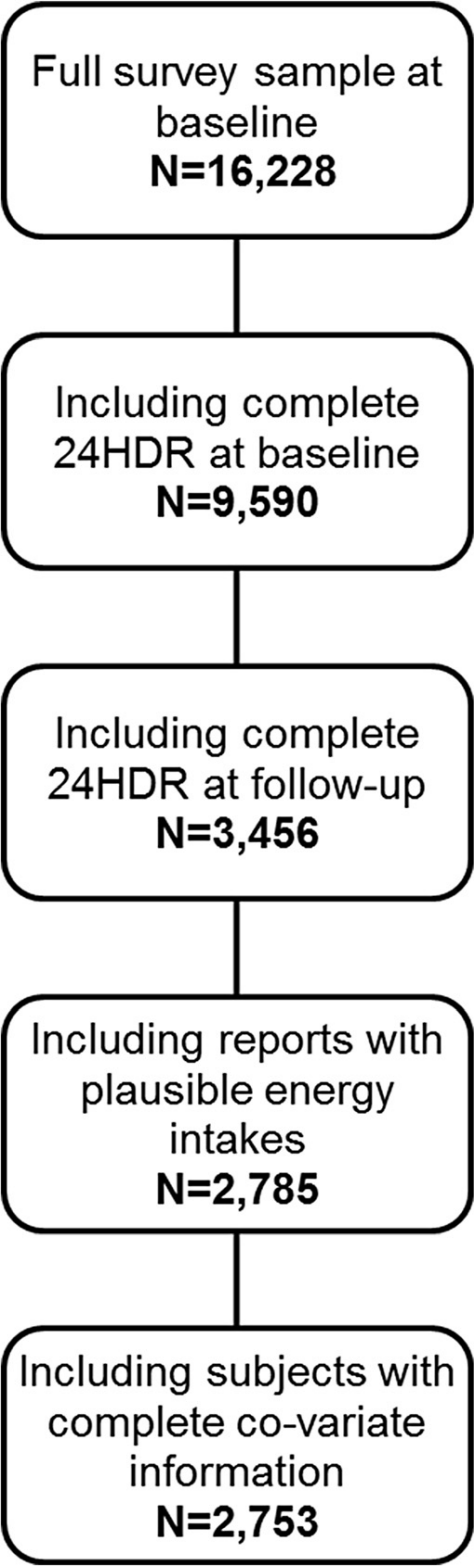
Inclusion criteria for study sample

From 16228 children who participated in the IDEFICS baseline examinations 9590 children provided at least one complete T0 24HDR. Among these, 3456 individuals also contributed at least one complete T1 24HDR, and 2785 had plausible reported EI values at T0 and T1. A total of 2753 children with complete co-variate information were eligible for the final analysis (study sample, see Fig. 1).

No substantial differences were observed when comparing main characteristics such as weight group, age and sex distribution or ISCED level of the baseline sample and the sample presented here (results not shown).

### Statistical analysis

Usual energy requirements and hence energy intakes differ depending on age, height and sex. To facilitate comparability of EI values of children of different ages, sex, and heights, EI values need to be adjusted. For this purpose, a growth model for EI during childhood dependent on age, height and sex was estimated. Typically, EI increases strongly during early childhood, to a smaller extent in later childhood and settles down in adulthood. To account for this non-linearity, the following fractional polynomial model for repeated measures (using T0 and T1 data simultaneously) was selected based on the Akaike information criterion to model EI growth during childhood:

$$ \begin{array}{l}E{I}_{i,j} = {\beta}_{\mathsf{0}} + {\beta}_{\mathsf{1}} ag{e}_{i,j}^{\mathsf{1}} + {\beta}_{\mathsf{2}} heigh{t}_{i,j}^{\mathsf{1}} + {\beta}_{\mathsf{3}} heigh{t}_{i,j}^{-\mathsf{1}} + {\beta}_{\mathsf{4}} heigh{t}_{i,j}^{-\mathsf{2}}+{\beta}_{\mathsf{5}}se{x}_i\\ {}\kern8em  + {\beta}_{\mathsf{6}} heigh{t}_{i,j}^{\mathsf{1}}\ast se{x}_i + {\beta}_{\mathsf{7}} heigh{t}_{i,j}^{-\mathsf{1}}\kern0.5em \ast \kern0.5em se{x}_i + {\beta}_{\mathsf{8}} heigh{t}_{i,j}^{-\mathsf{2}}\kern0.5em \ast \kern0.5em se{x}_i + {\varepsilon}_{i,j}\end{array} $$

(for individual i, i = 1,…,2757, at measurement occasion j, j = 1,2).

**Fig. 2:**
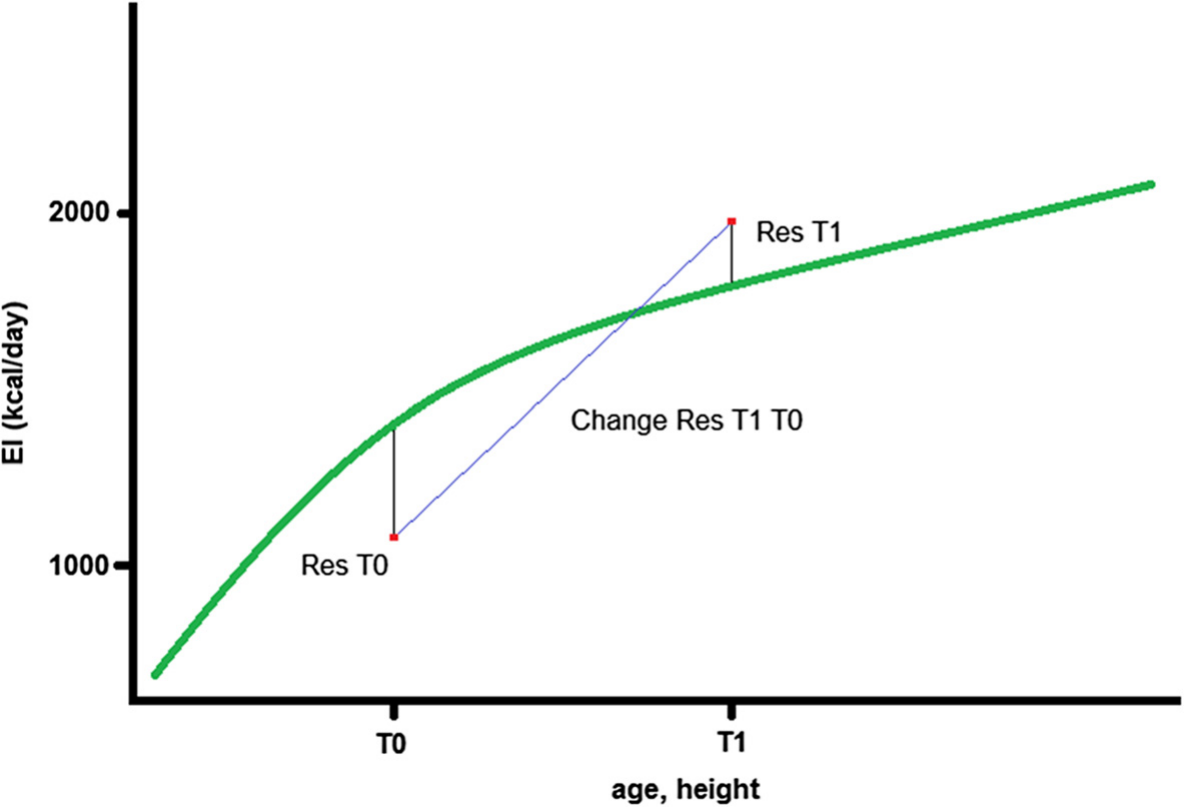
Visualization of an energy intake growth curve during childhood and exemplary display of residuals at T0 and T1 and change between the two time points. EI (kcal/day): energy intake (kcal/day); T0: baseline examinations; T1: follow-up examinations; Res T0: residuals of EI at baseline; Res T1: residuals of EI at follow-up; Change Res T1 T0: change in residuals between baseline and follow-up examination

The residual EI ${\varepsilon}_{i,j}$ indicates the subject-specific deviation from the average EI for a given age, height and sex as displayed in Fig. 2. For instance, a residual EI of 100 would indicate that this child consumed 100 kcal more than the average study population child with the same age, height and sex at this measurement occasion.

The estimated residuals for energy intake at T0 and T1 were used as exposure measures in the subsequent analyses. Residuals were rescaled such that one unit refers to 100 kcal in the regression models to achieve meaningful interpretations of effect estimates (1 unit ~ 100 kcal). In the longitudinal models, the annual change in residual EI (Δresidual EI) between T0 and T1 (Fig. 2) was calculated as exposure measure, i.e. Δresidual EI = (residual EI T1 – residual EI T0) / (age T1 – age T0).

In the cross-sectional analysis, the association between residuals of EI and BMI z-score at baseline was estimated using linear regression analysis. All models were adjusted for baseline age, sex and country (model 1). In a second model, baseline values of highest parental ISCED level, parental BMI and average screen time per week were additionally included (fully adjusted model 2). In a subgroup analysis (N = 1933, model 3), objectively measured PA as duration of MVPA (min/day) was added to model 2. To evaluate the longitudinal effect of Δresidual EI per year on ΔBMI z-score per year, linear regression models were fitted analogue to the models 1, 2 and 3 with additional adjustment for BMI z-score and residual EI at baseline.

As we found significant interactions between age as well as sex and residual EI in the above models, all models were fitted for the total study group as well as stratified by sex and by baseline age group (2- < 4 y, 4- < 6 y, 6- < 8 y and 8- < 10 y), e.g. children who were 2- < 4 y at baseline examination were assigned to the 2 to <4 y age group etc.

All analyses were performed using SAS® statistical software version 9.3 (SAS Institute, Inc., Cary, NC) using the procedure PROC MIXED.; 99 % confidence intervals are presented for ß_EI_ estimates in all models.

## Results

### Study sample

The study sample consisted of 48.2 % female participants; 67.2 % were normal weight with the highest prevalence in 2- < 4 y old children (75.1 %) compared to other age groups. Children from the study sample were more likely to be Italian (37.5 %), and less likely Belgian (3.3 %, Table 1).

**Table 1:** Characteristics of the study population (total group and stratified by age; total numbers and percentages)

	2- < 4 years		4- < 6 years		6- < 8 years		8- < 10 years		All	
	N	%	N	%	N	%	N	%	N	%
Country										
Italy	174	36.2	284	39	378	35.8	196	40.3	1032	37.5
Estonia	48	10	81	11.1	125	11.8	98	20.2	352	12.8
Cyprus	12	2.5	28	3.8	83	7.9	14	2.9	137	5
Belgium	26	5.4	33	4.5	30	2.8	2	0.4	91	3.3
Sweden	96	20	93	12.8	134	12.7	68	14	391	14.2
Germany	57	11.9	111	15.2	143	13.5	46	9.5	357	13
Hungary	28	5.8	41	5.6	89	8.4	51	10.5	209	7.6
Spain	40	8.3	58	8	75	7.1	11	2.3	184	6.7
ISCED-Level of parents^a^										
Low Education	44	9.1	69	9.5	86	8.1	52	10.7	251	9.1
Medium Education	250	52	421	57.8	582	55.1	284	58.4	1537	55.8
High Education	187	38.9	239	32.8	389	36.8	150	30.9	965	35.1
Sex of the child										
Boys	259	53.8	403	55.3	544	51.5	221	45.5	1427	51.8
Girls	222	46.2	326	44.7	513	48.5	265	54.5	1326	48.2
Weight group										
Underweight	55	11.4	85	11.7	92	8.7	40	8.2	272	9.9
Normal weight	361	75.1	491	67.4	699	66.1	300	61.7	1851	67.2
Overweight	52	10.8	98	13.4	171	16.2	92	18.9	413	15.0
Obese	13	2.7	55	7.5	95	9.0	54	11.1	217	7
Study region										
Intervention region	273	56.8	416	57.1	548	51.8	254	52.3	1491	54.2
Control region	208	43.2	313	42.9	509	48.2	232	47.7	1262	45.8
All	481	100	729	100	1057	100	486	100	2753	100

^a^ International Standard Classification of Education Maximum (ISCED); maximum of both parents (0, 1, 2 = low education; 3, 4 = medium education; 5, 6 = high education)

### Baseline and follow-up characteristics

On average, boys had higher usual intakes of energy (kcal/day) than girls and older children had higher usual intakes than younger children at baseline and at follow-up (Table 2). For pre-school children and especially for pre-school boys (2- < 4 y, 4- < 6 y), change in daily EI was higher than for school age children (6- < 8 y, 8- < 10 y). Body height (cm) was slightly higher for girls than for boys at T0 and follow - up examination. Across age-groups, change in height between T0 and T1 was highest in children aged 2- < 4 y at baseline and lowest in the oldest age group. Both boys and girls had a slight increase in relative BMI z-scores between baseline and follow-up examination, with boys having a higher increase than girls (+0.08 kg/m^2^ and +0.05 kg/m^2^ respectively). Screen time per week was higher for boys than for girls. Overall, mean parental BMI was similar for parents of boys and girls (24.20 kg/m^2^). Duration of MVPA per day was generally higher for boys than for girls.Baseline and follow-up characteristics of exposure and outcome variates differ for the subgroup of children (Additional file 1).

**Table 2:** Baseline and follow-up characteristics (mean and SD) of exposure and outcome variates given by sex and age group at baseline

		2- < 4 years	4- < 6 years	6- < 8 years	8- < 10 years	All
		boys = 259;	boys = 403;	boys = 544;	boys = 221;	boys = 1427;
		girls = 222	girls = 326	girls = 513	girls = 265	girls = 1326
		Mean (SD)	Mean (SD)	Mean (SD)	Mean (SD)	Mean (SD)
Daily energy intake (kcal/day) at baseline	boys	1423.1 (119.6)	1553.6 (125.9)	1706.1 (126.3)	1817.4 (135.2)	1628.9 (181.8)
	girls	1303.8 (108.3)	1429.9 (108.1)	1580.8 (111.5)	1669.6 (113.4)	1515.1 (167.0)
Daily energy intake (kcal/day) at follow-up	boys	1629.1 (211.4)	1764.7 (249.4)	1838.9 (272.7)	1912.6 (255.1)	1791.3 (268.5)
	girls	1514.0 (203.8)	1592.5 (221.1)	1707.1 (245.5)	1752.9 (252.1)	1655.8 (249.2)
Change in daily energy intake (kcal)	boys	206.0 (214.2)	211.2 (243.8)	132.8 (276.1)	95.2 (272.7)	162.4 (260.0)
	girls	210.2 (204.2)	162.7 (231.9)	126.4 (254.8)	83.3 (248.2)	140.7 (243.3)
Height (cm) at baseline	boys	99.5 (5.7)	110.9 (5.7)	124.9 (6.4)	132.6 (6.0)	117.5 (12.8)
	girls	98.4 (6.0)	109.7 (6.1)	124.3 (6.5)	131.7 (5.5)	117.9 (13.1)
Height (cm) at follow-up	boys	113.8 (5.9)	124.2 (5.9)	136.5 (6.9)	143.4 (6.5)	130.0 (11.9)
	girls	113.4 (6.5)	123.0 (6.2)	135.9 (7.1)	143.6 (6.7)	130.5 (12.4)
Change in height (cm)	boys	14.4 (2.6)	13.3 (1.9)	11.6 (2.0)	10.9 (2.0)	12.5 (2.4)
	girls	15.0 (2.3)	13.4 (2.4)	11.6 (2.3)	12.0 (3.0)	12.7 (2.7)
BMI z-score^a^ (kg/m^2^) at baseline	boys	0.17 (1.02)	0.27 (1.22)	0.51 (1.25)	0.57 (1.22)	0.39 (1.21)
	girls	0.13 (1.03)	0.51 (1.16)	0.54 (1.12)	0.64 (1.15)	0.48 (1.13)
BMI z-score^a^ (kg/m^2^) at follow-up	boys	0.38 (1.19)	0.56 (1.25)	0.59 (1.27)	0.59 (1.19)	0.55 (1.24)
	girls	0.37 (1.18)	0.66 (1.16)	0.59 (1.09)	0.64 (1.16)	0.58 (1.14)
Annual change in BMI z-score^a^ (kg/m^2^)	boys	0.11 (0.41)	0.15 (0.33)	0.04 (0.33)	0.01 (0.24)	0.08 (0.34)
	girls	0.12 (0.35)	0.08 (0.34)	0.03 (0.24)	0.0 (0.21)	0.05 (0.29)
Screen time (hours per week)	boys	8.8 (6.0)	12.0 (7.7)	13.6 (7.4)	16.3 (8.7)	12.7 (7.8)
	girls	7.7 (5.5)	10.2 (6.3)	11.8 (6.8)	12.7 (6.8)	10.9 (6.7)
Parental BMI	boys	24.32 (4.48)	24.44 (4.66)	24.07 (4.15)	23.97 (3.89)	24.20 (4.32)
	girls	24.18 (4.62)	24.45 (4.8)	23.97 (4.03)	24.31 (4.41)	24.20 (4.46)
MVPA (minutes per day)^b^; subgroup analysis^c^		2- < 4 years	4- < 6 years	6- < 8 years	8- < 10 years	All
		boys = 154;	boys = 283;	boys = 390;	boys = 177;	boys = 1004;
		girls = 133	girls = 222	girls = 374	girls = 200	girls = 929
	boys	36.2 (19.3)	42.5 (23.0)	44.7 (24.7)	42.0 (24.8)	43.1 (23.7)
	girls	28.6 (14.0)	33.8 (18.4)	34.2 (17.4)	34.2 (20.7)	33.3 (18.0)

^a^BMI z-scores according to Cole et al. [22]

^b^Duration MVPA according to Evenson (Trost et al. [23])

^c^Baseline and follow-up characteristics of exposure and outcome variates differ for the subgroup of children (Additional file 1)

### Cross-sectional effect of residuals of energy intake at baseline on baseline BMI z-score

For the total study sample, for boys as well as for 6- < 8 y and 8- < 10 y olds positive associations were observed between residuals of EI and baseline BMI z-score in the crude and adjusted models (models 1 and 2) and except for the 6- < 8 y olds also in model 3 with additional adjustment for MVPA (Table 3). Association was strongest in 8- < 10 y olds (ß_EI_ 0.14, 99%CI 0.23 – 0.26, model 3). This indicates that a child consuming 100 kcal/day more than the average study population child with the same age, height and sex at baseline had a 0.14 kg/m^2^ higher BMI z-score at baseline. No association was found for girls, and in the younger age groups (2- < 4 y olds and 4- < 6 y olds).

**Table 3:** Cross-sectional effect of age, height and sex-adjusted residuals of energy intake at baseline (1unit ~ 100 kcal) on BMI z-score at baseline stratified by sex and age group at baseline

	Boys		Girls		2- < 4 years		4- < 6 years		6- < 8 years		8- < 10 years		All	
	(N = 1427)		(N = 1326)		(N = 481)		(N = 729)		(N = 1057)		(N = 486)		(N = 2753)	
	ß_EI_	SE	ß_EI_	SE	ß_EI_	SE	ß_EI_	SE	ß_EI_	SE	ß_EI_	SE	ß_EI_	SE
	99 % CI		99 % CI		99 % CI		99 % CI		99 % CI		99 % CI		99 % CI	
**Model 1**	**0.11**	**0.03**	0.06	0.03	-0.02	0.05	0.07	0.04	**0.11**	**0.03**	**0.15**	**0.04**	**0.09**	**0.02**
	**0.04 - 0.18**		-0.02 - 0.13		-0.14 - 0.10		-0.04 - 0.18		**0.03 - 0.19**		**0.04 - 0.25**		**0.04 - 0.14**	
**Model 2**	**0.11**	**0.03**	0.06	0.03	-0.01	0.05	0.08	0.04	**0.10**	**0.03**	**0.12**	**0.04**	**0.09**	**0.02**
	**0.04 - 0.18**		-0.01 - 0.14		-0.13 - 0.10		-0.02 - 0.19		**0.02 - 0.18**		**0.02 - 0.22**		**0.04 - 0.14**	
**Model 3**	**0.13**	**0.03**	0.04	0.03	-0.02	0.06	0.08	0.05	0.08	0.04	**0.14**	**0.05**	**0.09**	**0.20**
	**0.05 - 0.21**		-0.05 - 0.12		-0.18 - 0.13		-0.04 - 0.21		-0.02 - 0.17		**0.02 - 0.26**		**0.03 - 0.15**	

Model 1) is the crude model adjusted for age, sex, country, if not stratified by age/sex, respectively

Model 2) moreover includes maximum parental ISCED level, parental BMI and screen time

Model 3) moreover includes MVPA (subgroup analysis; N = 1, 933)

(Models stratified by sex are not adjusted for sex)

*ß*_*EI*_ Estimate for EI, *SE* Standard Error, *99 % CI* 99 % Confidence Interval

Further, adjusting for residing in the intervention vs. control region did not change findings (data not shown).

### Longitudinal effect of residual change in energy intake per year on ΔBMI z-score per year

For the total study sample and for 4- < 6 y olds positive associations were observed between Δresidual EI per y on ΔBMI z-score per y in the crude model 1 and the fully adjusted model 2 (Table 4). Associations were strongest in 4- < 6 y olds. No association was found for girls, 2- < 4 y olds, 6- < 8 and 8- < 10 y olds. Overall, size of the effect estimate was smaller longitudinally, whereas association was stronger cross-sectionally. Adjusting for residing in the intervention vs. control region did not change findings (data not shown).

**Table 4:** Longitudinal effect of change in residual energy intake per year (Δresiduals EI, 1unit ~ 100 kcal) on ΔBMI z-score per year stratified by sex and baseline age group

	Boys		Girls		2- < 4 years		4- < 6 years		6- < 8 years		8- < 10 years		All	
	(N = 1427)		(N = 1326)		(N = 481)		(N = 729)		(N = 1057)		(N = 486)		(N = 2753)	
	ß_EI_	SE	ß_EI_	SE	ß_EI_	SE	ß_EI_	SE	ß_EI_	SE	ß_EI_	SE	ß_EI_	SE
	99 % CI		99 % CI		99 % CI		99 % CI		99 % CI		99 % CI		99 % CI	
**Model 1**	0.017	0.007	0.011	0.007	0.013	0.017	**0.029**	**0.010**	0.006	0.007	0.001	0.008	**0.014**	**0.005**
	-0.002 - 0.036		-0.006 - 0.029		-0.031 - 0.057		**0.003 - 0.057**		-0.013 - 0.025		-0.020 - 0.023		**0.001 - 0.027**	
**Model 2**	0.0178	0.007	0.011	0.007	0.021	0.017	**0.032**	**0.010**	0.005	0.007	0.000	0.008	**0.015**	**0.005**
	-0.001 - 0.036		-0.006 - 0.028		-0.023 - 0.065		**0.005 - 0.058**		-0.014 - 0.024		-0.021 - 0.021		**0.002 - 0.027**	
**Model 3**	0.015	0.008	0.006	0.008	0.024	0.020	0.027	0.012	0.003	0.009	-0.003	0.009	0.011	0.005
	-0.006 - 0.037		-0.014 - 0.025		-0.029 - 0.078		-0.003 - 0.058		-0.019 - 0.026		-0.028 - 0.022		-0.003 - 0.026	

Model 1) is the crude model adjusted for BMI z-score and residual EI at baseline, country, age, sex, if not stratified by age/sex, respectively

Model 2) moreover includes maximum parental ISCED level, parental BMI and screen time

Model 3) moreover includes MVPA (subgroup analysis; N = 1, 933)

(Models stratified by sex are not adjusted for sex)

*ß*_*EI*_ Estimate for EI, *SE* Standard Error, *99 % CI* 99 % Confidence Interval

In sensitivity analyses, the longitudinal associations were additionally estimated relating the (1) change in EI per cm increase in height (ΔEI/Δheight) and (2) change in usual EI (ΔEI) to ΔBMI z-score per y adjusting for baseline values of EI and BMI z-score. These exposure measures are easier to interpret, but less precise with respect to the age- and height dependencies of energy intake such that we finally decided to present the estimates based on residual changes. However, using ΔEI/Δheight or ΔEI as exposures, results were similar to those described above: Again significant positive associations were found for the total study group, in boys and in the group of 4- < 6 y olds.

## Discussion

Cross-sectionally, we observed positive associations between residual EI and BMI z-score for the full study sample and in older (≥6 years) but not in younger children. Longitudinally, small positive associations were observed between Δresidual EI per y on ΔBMI z-score per y for the full study sample and in 4- < 6 y old children.

Previous studies have assessed longitudinal changes in diet assuming a linear change between ages [10, 11, 30]. Anderson [34] for the first time modelled a piecewise linear relationship between age and energy intake to account for greater increases of EI in earlier childhood (from 3 - 7 y) compared to later childhood (7 -13 y). Findings from the present study confirmed an overall non-linear increase in daily EI during childhood depending on changes in height, sex and age. Changes in daily EI (from baseline to follow-up) decline non-linearly across age groups, especially for boys who at the same time show a decrease in change of height growth (Table 2). Using residual EI we accounted for non-linearity in changes in energy intake during childhood in this sample of pre-school children and school aged children.

The European Society for Paediatric Gastroenterology, Hepatology, and Nutrition (ESPGHAN) Committee on Nutrition supports the prevention of childhood obesity and recommends the individual determination of energy intake, taking energy expenditure and growth into account [35]. In our study, results from the cross-sectional analyses revealed associations between residual EI at baseline and baseline BMI z-score for the whole study sample, boys and 6- < 8 y and 8- < 10 y olds. These findings support previous associations between BMI z-score and total EI in school children from other cross-sectional studies [5, 6, 36]. Cross-sectional observations provide no insight into temporality of the relationship between EI and BMI z-score, and reverse causation is considered a major concern. Our longitudinal findings provide better evidence to support causality between EI and subsequent changes in BMI z-score, even though size of the effect estimate was larger cross-sectionally. Results from the present longitudinal analyses showed that Δresidual EI was associated with subsequent increase in annual BMI z-score for the full study sample and for 4- < 6 y olds. These findings are both, in agreement [14, 30, 37] and disagreement [9, 11] with results from other longitudinal studies investigating the association between EI with subsequent change in BMI z-score. In our study, reported EI was slightly lower than recommended by the Scientific Committee on Food for boys and girls in the European Community [38]. We did not find associations between residual EI and BMI z-score for girls, neither cross-sectionally nor longitudinally. For girls, changes in EI and relative ΔBMI z-score were generally lower than for boys, especially for the 4- < 6 y olds. Higher EI for boys than for girls has been observed in previous longitudinal European and U.S. investigations [11, 30, 39]. The observed higher consumption of energy-dense foods among growing children may arise from the necessity to fulfil increased energy demands [40]. In contrast to earlier longitudinal studies [14, 39], changes in EI among 4- < 6 y boys of our sample was higher while their change in height was lower (compared to girls of the same age with lower change in EI, Table 2) and may explain the subsequent increase of change in BMI z-scores. Even though cross-sectional data suggest that for boys, an EI above the average EI for the respective age and height may be an important determinant of increases in BMI z-score two years later this association was not observed in the longitudinal analysis.

Longitudinal findings from earlier research describe the protective role of physical activity on weight gain, implying an important role of physical activity in the development of obesity [41–43]. In U.S. children and adolescents, MVPA was inversely associated with fat mass and with BMI [8]. In our study, longitudinally Δresidual EI was no longer associated with ΔBMI z-score after adjustment for daily minutes of MVPA in a subsample of the children. However, the size of the effect estimate remained almost unchanged such that the insignificant association might be a result of the smaller power resulting from the smaller sample size. Our finding underscores the importance of studying sex- and age-related energy intakes, as they are influenced by growths and most likely by PA in seeking to understand the complex relationships between utilization (growth, expenditure or storage) of dietary energy and BMI z-score in childhood.

### Strengths and limitations

In the IDEFICS study dietary information was given by proxy-respondents. Proxy-reporting relates to the number of meals under parental control; incomplete reporting of dietary intakes may contribute to reporting bias [44]. In order to reduce errors due to portion size estimation, erroneous food composition tables, incomplete recalls, misreporting or daily variations in intake we followed a rigorous approach. Firstly, the development of a computer-assisted assessment tool with standardized photographs, with multiple plausibility checks and reminding questions facilitated reporting of accurate portion sizes and complete recalls. School/kindergarten meal assessment through observers helped to complete all foods and beverages consumed in the setting. Secondly, the exclusion of incomplete recalls and recalls with implausible energy reporting corrected substantially for reporting bias. Plausible reporters (mean age 8.4 y) were found to be older than over-reporters (7.3 y) and younger than under-reporters (8.7 y); the mean BMI z-score (0.72 kg/m^2^) of plausible reporters was higher than that of over-reporters (+0.12 kg/m^2^) and lower than that of under-reporters (1.44 kg/m^2^) as observed in earlier studies [13]. Hence, a small selection bias that may have led to an attenuation of effect estimates cannot completely be precluded. However, inclusion of misreports may obscure or even inverse relationships with weight status as recently reported [45]. Finally, deriving the usual EI based on the NCI method [29] and accounting for day-to-day variation in EI is a clear strength of this study. Even though proxy-reported dietary data has limitations, we still believe our data is valuable for population research.

The IDEFICS study allows a deeper insight into the effect of deviations from usual age-, sex- and height-specific EI on BMI z-scores in 2 to 10 y old children across Europe. The calculation of residual EI as exposure variable instead of merely using EI as done in previous studies, helped to account for growth-related changes in usual EI when investigating the effect of EI on BMI z-scores in a population of children covering a wide age span. However, using residuals from a growth model as exposure induces uncertainty in the effect estimates and may hence increase the risk for type 2 error. In addition, no objective measurements of individual energy expenditure were available for the two examination time points (and PA only in a sub-group). Hence, residual EI estimates did account for differences in EI resulting not only from differences of body height, height growth but also from different PA levels between children. In a “perfect” data situation, the best option for the estimation of residual EI might be to regress EI on measured total energy expenditure, but unfortunately measuring total energy expenditure using doubly labeled water (DLW) was not feasible in this large-scale multi-centre children cohort for cost, logistic and ethical reasons.

In the present study, no substantial differences were observed when comparing main characteristics of the baseline sample and the sample presented here. As only 17 % of the initial baseline cohort provided follow-up data and complete co-variate information, the IDEFICS Study - like many cohort studies - may therefore suffer from volunteer bias; we acknowledge this as a possible study limitation. However, the large sample size comprises data from eight European countries; the strictly standardized data assessment, documentation and data cleaning processing guarantee the highest possible data quality. Further, availability of relevant confounders, such as parental BMI and educational level, screen time and objectively measured MVPA is an additional strength of this study.

## Conclusion

In conclusion, age-, sex-, and height-specific deviations from population average EI were weakly associated cross-sectional and longitudinal with changes in BMI z-scores in European children aged 2 to 10 y old. Longitudinal associations between changes in EI and BMI were particularly observed for the full study sample and for 4- < 6 y old children. Our results suggest that the promotion of appropriate energy intakes may be most effective when considering sex-and age-related energy demands due to different growth and PA levels according to the recommendation of the ESPGHAN Committee on Nutrition. Residual EI seems to be a useful exposure measures when studying effects of dietary exposures in children with a wide age span as they accommodate the growth-related changes in dietary intake during childhood.

## Additional file

Additional file 1:
**Baseline and** **follow-up** **characteristics (mean and SD) of exposure and outcome variates given by sex and age group at baseline for** **MVPA-subgroup.** (DOCX 21 kb)

## Abbreviations

BMI: body mass index
DLW: doubly labeled water
EI: energy intake
ESPGHAN: European Society for Paediatric Gastroenterology, Hepatology, and Nutrition
FCT: food composition tables
IDEFICS Study: Identification and prevention of Dietary- and lifestyle-induced health EFfects In Children and infantS Study
ISCED: International Standard Classification of Education
MVPA: moderate to vigorous physical activity
NCI: National Cancer Institute (USA)
PA: physical activity
SACINA: Self-Administered Children and Infant Nutrition Assessment’
SES: Socio economic status
T0/T1: Baseline examinations/follow-up
TV: television
24HDR: 24-hr dietary recalls
