# Supplementary material for: Cross-sectional and longitudinal associations between energy intake and BMI z-score in European children
Source: Int J Behav Nutr Phys Act. 2016 Feb 16;13:23. doi: 10.1186/s12966-016-0344-3 (PMC4754870; doi:10.1186/s12966-016-0344-3)
Supplement: Additional file 1: — Baseline and follow-up characteristics (mean and SD) of exposure and outcome variates given by sex and age group at baseline for MVPA-subgroup. (DOCX 21 kb) [file 12966_2016_344_MOESM1_ESM.docx]

(ONLINE SUPPLEMENTAL MATERIAL)

Baseline and follow-up characteristics (mean and SD) of exposure and outcome variates given by sex and age group at baseline for MVPA-subgroup

|  | | 2-<4 years  boys=154;  girls=133 | 4-<6 years  boys=283;  girls=222 | 6-<8 years  boys=390;  girls=374 | 8-<10 years  boys=177;  girls=200 | All  boys=1004;  girls=929 |
| --- | --- | --- | --- | --- | --- | --- |
|  |  | **Mean (SD)** | **Mean (SD)** | **Mean (SD)** | **Mean (SD)** | **Mean (SD)** |
| **Daily energy intake (kcal/day) at baseline** | boys | 1442.8 (115.7) | 1551.4 (123.1) | 1712.3 (127.4) | 1816.9 (131.1) | 1644.0 (177.0) |
|  | girls | 1315.3 (107.3) | 1429.6 (100.8) | 1584.9 (112.9) | 1669.0 (115.5) | 1527.3 (162.6) |
| **Daily energy intake (kcal/day) at follow-up** | boys | 1638.6 (223.5) | 1778.5 (255.8) | 1858.2 (269.5) | 1914.4 (263.5) | 1812.0 (272.0) |
|  | girls | 1518.0 (200.8) | 1617.9 (219.4) | 1723.6 (243.9) | 1746.9 (251.8) | 1673.9 (247.2) |
| **Change in daily energy intake (kcal)** | boys | 195.8 (228.6) | 227.2 (250.1) | 145.9 (278.7) | 97.6 (279.4) | 167.9 (267.5) |
|  | girls | 202.6 (201.0) | 188.2 (224.3) | 138.6 (252.2) | 77.9 (251.9) | 146.6 (242.5) |
| **Height (cm) at baseline** | boys | 99.9 (5.7) | 111.1 (5.5) | 125.1 (6.4) | 132.4 (5.9) | 118.6 (2.5) |
|  | girls | 99.2 (6.0) | 109.7 (6.0) | 124.5 (6.4) | 131.8 (5.6) | 118.9 (12.7) |
| **Height (cm) at follow-up** | boys | 114.4 (6.0) | 124.4 (5.8) | 136.8 (6.8) | 143.3 (6.6) | 131.0 (11.6) |
|  | girls | 113.9 (6.7) | 123.0 (6.0) | 136.2 (6.9) | 143.8 (6.8) | 131.5 (12.1) |
| **Change in height (cm)** | boys | 14.6 (2.8) | 13.3 (2.0) | 11.7 (1.8) | 10.9 (2.1) | 12.4 (2.4) |
|  | girls | 14.7 (2.2) | 13.3 (2.4) | 11.7 (2.1) | 12.0 (2.6) | 12.6 (2.6) |
| **BMI z-score^1^ (kg/m²) at baseline** | boys | 0.24 (0.99) | 0.2 (1.19) | 0.55 (1.26) | 0.57 (1.24) | 0.41 (1.21) |
|  | girls | 0.07 (1.00) | 0.49 (1.11) | 0.5 (1.1) | 0.64 (1.15) | 0.46 (1.11) |
| **BMI z-score^1^ (kg/m²) at follow-up** | boys | 0.45 (1.22) | 0.44 (1.2) | 0.61 (1.13) | 0.59 (1.2) | 0.53 (1.25) |
|  | girls | 0.29 (1.07) | 0.64 (1.16) | 0.53 (1.08) | 0.63 (1.18) | 0.55 (1.12) |
| **Annual change in BMI z-score^1^ (kg/m²)** | boys | 0.20 (0.74) | 0.24 (0.64) | 0.06 (0.67) | 0.02 (0.48) | 0.13 (0.65) |
|  | girls | 0.22 (0.64) | 0.15 (0.62) | 0.04 (0.45) | -0.01 (0.41) | 0.08 (0.52) |
| **Parental BMI** | boys | 24.40 (4.80) | 24.32 (4.54) | 24.36 (4.19) | 23.94 (3.89) | 24.28 (4.33) |
|  | girls | 24.36 (4.69) | 24.38 (4.97) | 23.81 (3.96) | 24.55 (5.00) | 24.18 (4.56) |
| **Screen Time (hours per week)** | boys | 8.8 (5.4) | 12.0 (7.8) | 13.5 (7.7) | 16.4 (9.0) | 12.9 (8.0) |
|  | girls | 7.9 (6.0) | 10.3 (6.2) | 11.5 (6.2) | 12.6 (6.6) | 10.9 (6.4) |
| **MVPA (minutes per day)^2^** | boys | 36.2 (19.3) | 42.5 (23.0) | 44.7 (24.7) | 42.0 (24.8) | 43.1 (23.7) |
|  | girls | 28.6 (14.0) | 33.8 (18.4) | 34.2 (17.4) | 34.2 (20.7) | 33.3 (18.0) |

^1^ Weight categories according to Cole et al. 2012

2 Duration MVPA according to Evenson (Trost et al. 2011)
